# Supplementary material for: Dynamics of history-dependent perceptual judgment
Source: Nat Commun. 2021 Oct 15;12:6036. doi: 10.1038/s41467-021-26104-2 (PMC8521591; doi:10.1038/s41467-021-26104-2)
Supplement: Supplementary file 1 — Supplementary Information [file 41467_2021_26104_MOESM1_ESM.pdf]

Supplementary Materials - **Dynamics of history-dependent perceptual judgment**

Hachen I., Reinartz S., Brasselet R., Stroligo A., Diamond M.E.

Supplementary Figures:

S1. Evolution of performance during the first 100 trials and illustration of the number of trials needed to infer a significant separation between PSEs in different stimulus ranges.

S2. Gradual psychometric function shift, excluding trials with incorrect *n-1* choice, suggests that *n-1* Δspeed drives the repulsive bias.

S3. Psychometric function parameters given *n*-1 choice and trial outcome.

S4. Psychometric function shift and PSE regression slope, excluding trials with correct *n-1* choice.

S5. The effect of stimulus *n-1* on psychometric function parameters and classification of stimulus *n* indicates mainly horizontal curve shift.

S6. Effect of ITI on history-dependent psychometric functions.

S7. Stimulus-dependent bias builds up between trials even when removing incorrect trials and considering only early-session trials. ITI is independent of Δspeed of stimulus *n-1*.

S8. Vibrotactile categorization task transferred to human tactile perception.

S9. Attractive choice bias in humans does not decay over the given ITI durations and does not explain the stimulus history bias after residualization.

S10. Comparative performance of the continuous and discretized history models with Area Under the ROC-curve (AUROC).

Supplementary Tables:

T1. Coefficients of logistic regression analysis including an interaction term between *n-1* stimulus and *n-1* reward.

T2. Coefficients of logistic regression analysis with interaction terms between the effect of stimulus *n-2* and *n-1* with the adjacent *ITIs*.

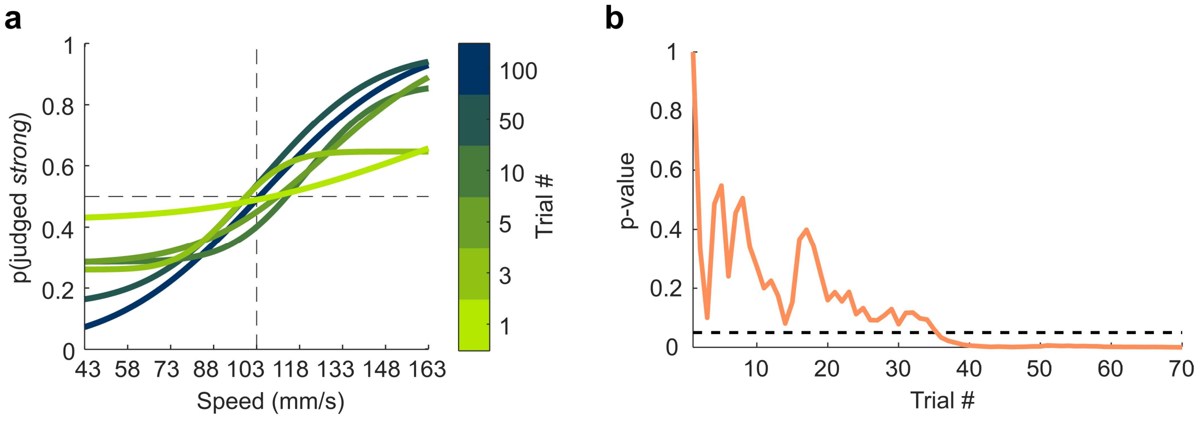

Supplementary Figure S1. Evolution of performance during the first 100 trials and illustration of the number of trials needed to infer a significant separation between PSEs in different stimulus ranges. a. Probability of categorizing stimuli as "strong" as a function of speed, with a stable range and boundary. Psychometric functions were fitted averaging data across all rats (corresponding to Figure 2a) from designated single trials at different stages of the session. **b.** Rank-sum tests between PSE values in the "high range" and in the "low range" conditions, based on trials from start of session to Trial # indicated on the abscissa. For each condition and each individual trial, a single PSE value was computed, averaging across sessions. Thus, for "high range" sessions the PSE was computed considering only trial 1, only trial 2, and so on until the last trial available; the same procedure was carried out for the "low range" sessions. The PSEs were then grouped cumulatively up to a given Trial # and tested for their difference between conditions. For example, for Trial # = 10, the 10 different PSEs computed from the first 10 trials of the "high range" were compared to the 10 different PSEs computed from the first 10 trials of the "low range". The p-values obtained by comparing the two groups of PSEs for each Trial # are reported on the ordinate, up to Trial # = 70. The dashed line denotes p-value = 0.05. Source data are provided as a Source Data file.

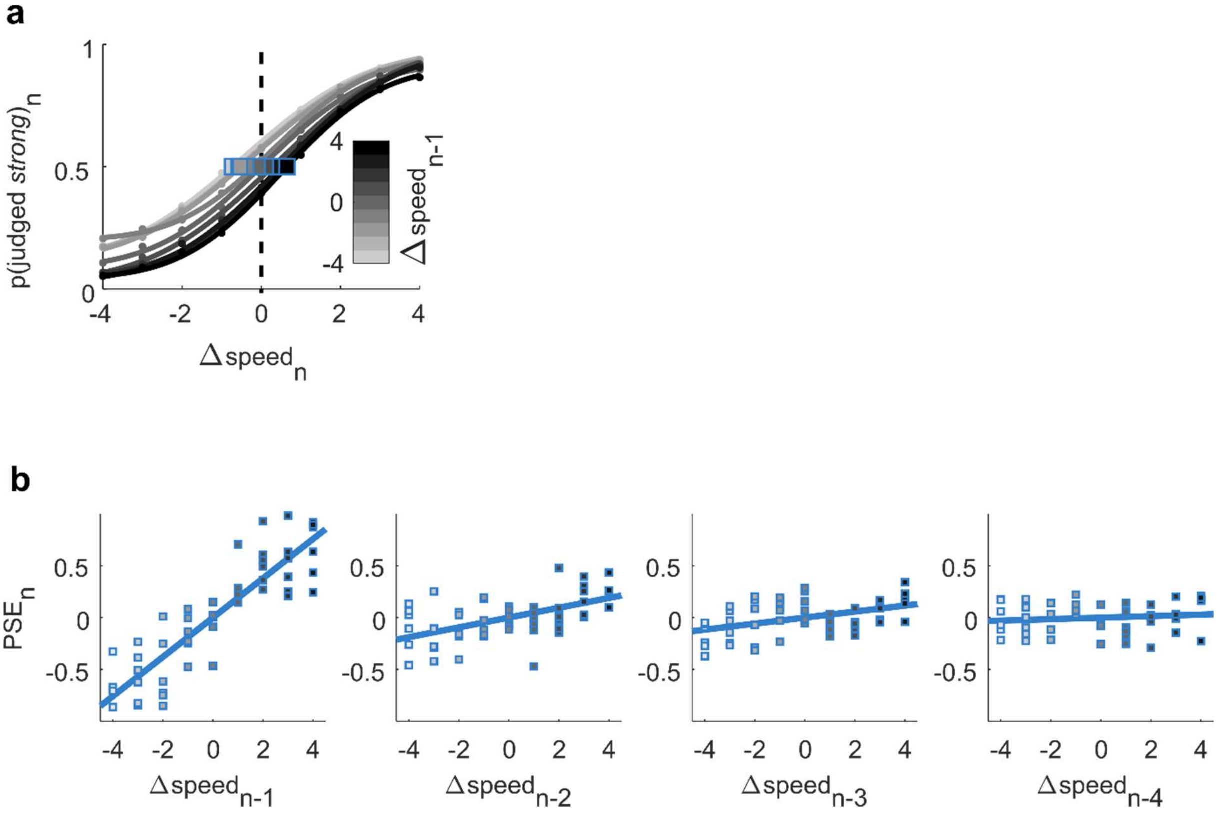

Supplementary Figure S2. Gradual psychometric function shift, excluding trials with incorrect *n-1* choice, suggests that *n-1* Δspeed drives the repulsive bias. a. Probability of categorizing stimulus *n* as "strong" as a function of trial *n* Δspeed, with curves grouped by Δspeed of trial *n-1,* only using correct choices in trial *n-1*. Darker curves correspond to higher *n-1* Δspeed. Blue squares denote PSE. **b.** Bias of the trial *n* psychometric curve, depending on Δspeed from trial *n-1* (far left plot) to trial *n-4* (far right plot), after removing incorrect trials in the corresponding trial *n-1* to trial *n-4*. Shading of the squares denotes Δspeed in trial *n-1*. Squares correspond to 6 individual rats. Source data are provided as a Source Data file.

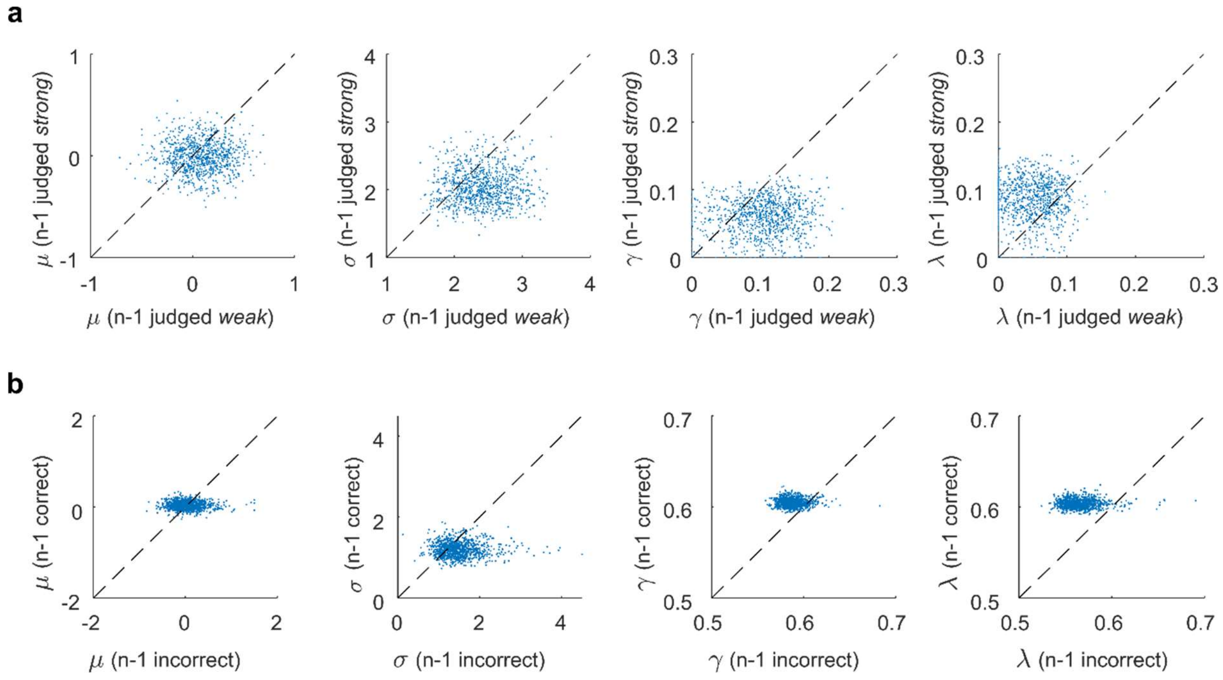

Supplementary Figure S3. Psychometric function parameters given *n-1* choice and trial outcome. a. Distributions of parameters obtained by bootstrapping the psychometric functions grouped by *n-1* choice (same data as shown in Fig. 3b). Trials were sampled with replacement (n = 1000), and both curves (*n-1* judged *weak*, *n-1* judged *strong*) were estimated for each artificial sample. A null difference could not be rejected for any parameter (90% confidence level). **b.** Distributions of parameters obtained by bootstrapping the psychometric functions grouped by trial *n -1* outcome (shown in Fig. 3c). Trials were sampled with replacement (n = 1000), and both curves (*n-1* incorrect*,* *n-1* correct) were estimated for each artificial sample. Confidence intervals around the resampled parameters did not exclude a null difference in *midpoint* or *slope* (confidence level < 90%), however it evidenced a difference in the asymptote parameters. Both lapse parameters, γ (lower lapse rate) and λ (upper lapse rate) slightly decreased after an incorrect trial (confidence = 92%, 97%, respectively), corresponding to an increase in lapse. Source data are provided as a Source Data file.

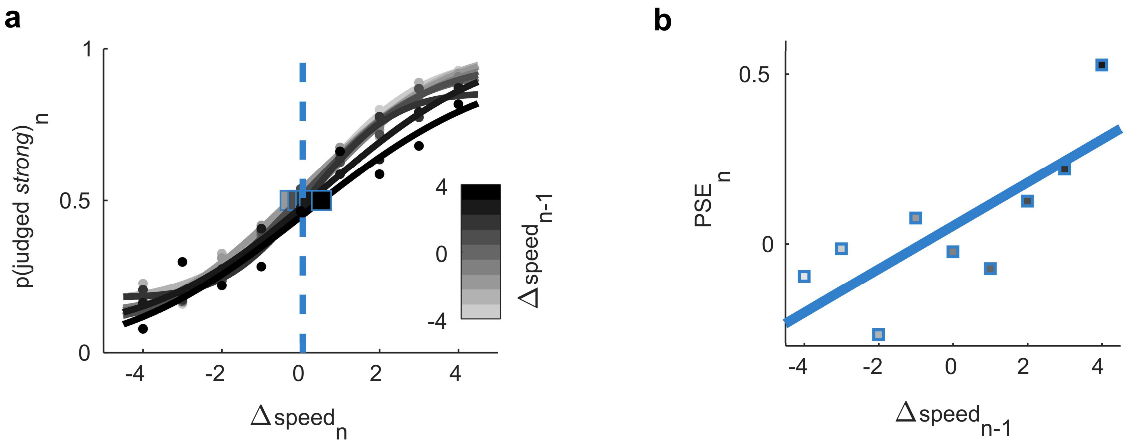

Supplementary Figure S4. Psychometric function shift and PSE regression slope, excluding trials with correct *n-1* choice. a. Probability of categorizing stimulus *n* as "strong" as a function of trial *n* Δspeed, with curves grouped by Δspeed of trial *n-1,* only using incorrect choices in trial *n-1*. Darker curves correspond to higher *n-1* Δspeed. Blue squares denote PSE. **b.** Bias of the trial *n* psychometric curve, depending on Δspeed in trial *n-1*, after removing correct trials in trial *n-1*. Shading of the squares denotes Δspeed in trial *n-1*. Squares corresponds to data, grouping all 6 rats. Source data are provided as a Source Data file.

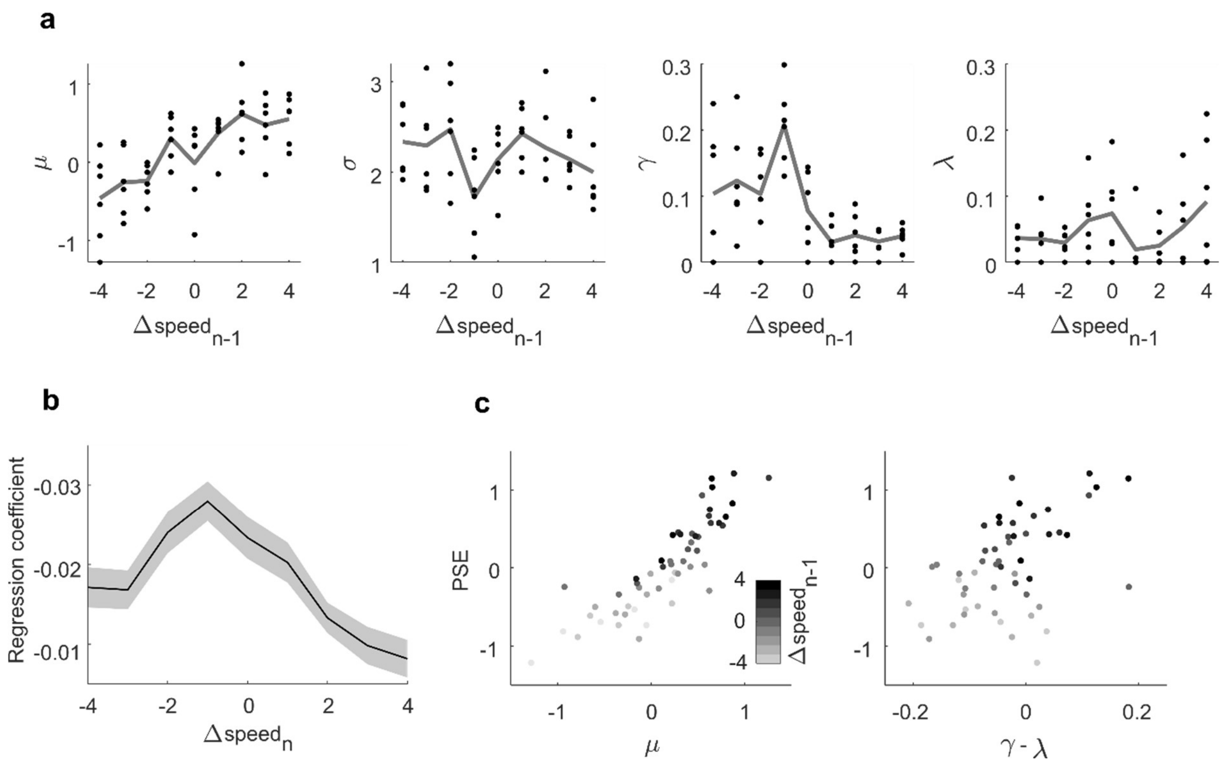

Supplementary Figure S5. The effect of stimulus *n-1* on psychometric function parameters and classification of stimulus *n* indicates mainly horizontal curve psychometric shift. a. Effect of Δspeed in trial *n-1* on psychometric curve parameters (curves, see Fig. 3a). Lower lapse rate (γ) was marginally modulated as a function of trial *n-1* Δspeed ($p=0.036$, two-tailed t-test of the correlation coefficient), while slope (σ) and upper lapse rate (λ) were not. The parameter most strongly influenced ($p<0.001$, two-tailed t-test of the correlation coefficient) by previous stimulus was the curve midpoint (μ), confirming horizontal shift as the psychometric curve property most influenced by the previous trial. **b.** Slopes of linear fits for p(judged "strong") regressed on Δspeed in trial *n*-1, for each trial *n* stimulus value. The resulting "bell" shape, with greater regression slopes for values of Δspeed near 0 indicates a greater effect of the previous stimulus on the evaluation of middle Δspeed values than on extreme Δspeed values. Transparent shading represents standard deviation of the bootstrapped regression coefficients. **c.** Change in PSE as a function of curve midpoint μ (left panel, R2 = 0.7422) and PSE versus combined lapse rate γ–λ (right panel, R2 = 0.2242). Gray scale denotes Δspeed in trial *n-1.* Only previous correct trials were considered (lapses may change depending on previous error). Source data are provided as a Source Data file.

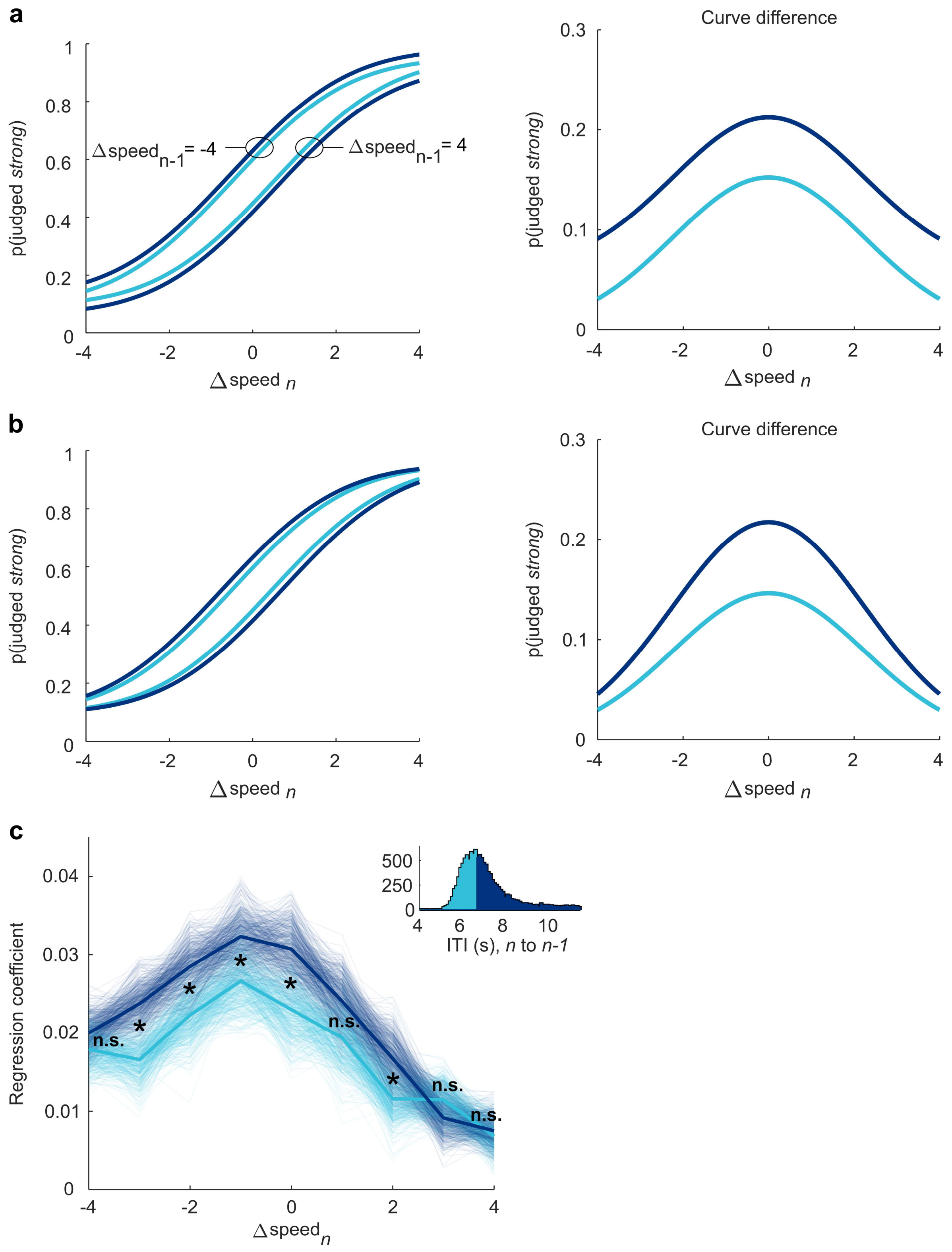

Supplementary Figure S6. Effect of ITI on history-dependent psychometric functions. a. Qualitative predictions for a history-dependent change in lapse rate modulated by ITI duration. Left: Two pairs of history-dependent psychometric curves following a short ITI (light blue) and long ITI (dark blue). The two pairs of curves have the same history-dependent midpoint

parameters, but asymmetrically different lapse rates: after a long ITI, the curves undergo a vertical shift in the opposite direction of the previous stimulus. Right: Lines represent the absolute difference between each pair of psychometric curves shown in the left plot. A history-dependent change in lapse rate modulated by ITI duration would lead to a constant separation between the two lines, meaning that the effect of the ITI is linearly combined with the effect of the previous stimulus **b.** Qualitative predictions for a history-dependent change in midpoint, but *not* lapse rates, modulated by ITI duration. Left: Two pairs of history-dependent psychometric curves following a short ITI (light blue) and long ITI (dark blue). The two pairs of curves have different history-dependent midpoint parameters and same lapse rates: after a long ITI, the curves undergo a larger horizontal shift in the opposite direction of the previous stimulus. Right: Lines represent the absolute difference between each pair of psychometric curves shown in the left plot. A history-dependent change in midpoint modulated by ITI duration would lead to a larger separation between the two lines towards the center of the stimulus range, and a smaller separation towards the extremes. In other words, the effect of the ITI interacts with the effect of the previous stimulus, amplifying the horizontal shift. **c.** Rats' data support the model of **b**, a history-dependent change in midpoint modulated by ITI duration. Similarly, as in Supplementary Figure S5b, the slope of the regression line for each combination of stimulus *n* and stimulus *n*-1 (9x9) was computed, this time separately after short and long ITI. A larger separation between the two lines towards the center of the stimulus range, and a smaller separation towards the extremes suggests that ITI durations modulate the history-dependent change in midpoint, corresponding with a largely horizontal shift of the psychometric curves. Two paired distributions of regression slopes (short ITI vs. long ITI) were obtained by bootstrapping rats' data. For each sample, we computed the difference between the two regression slopes in order to obtain a confidence interval. For each stimulus *n*, an asterisk indicates that the 90% confidence interval was excluding a null difference. Source data are provided as a Source Data file.

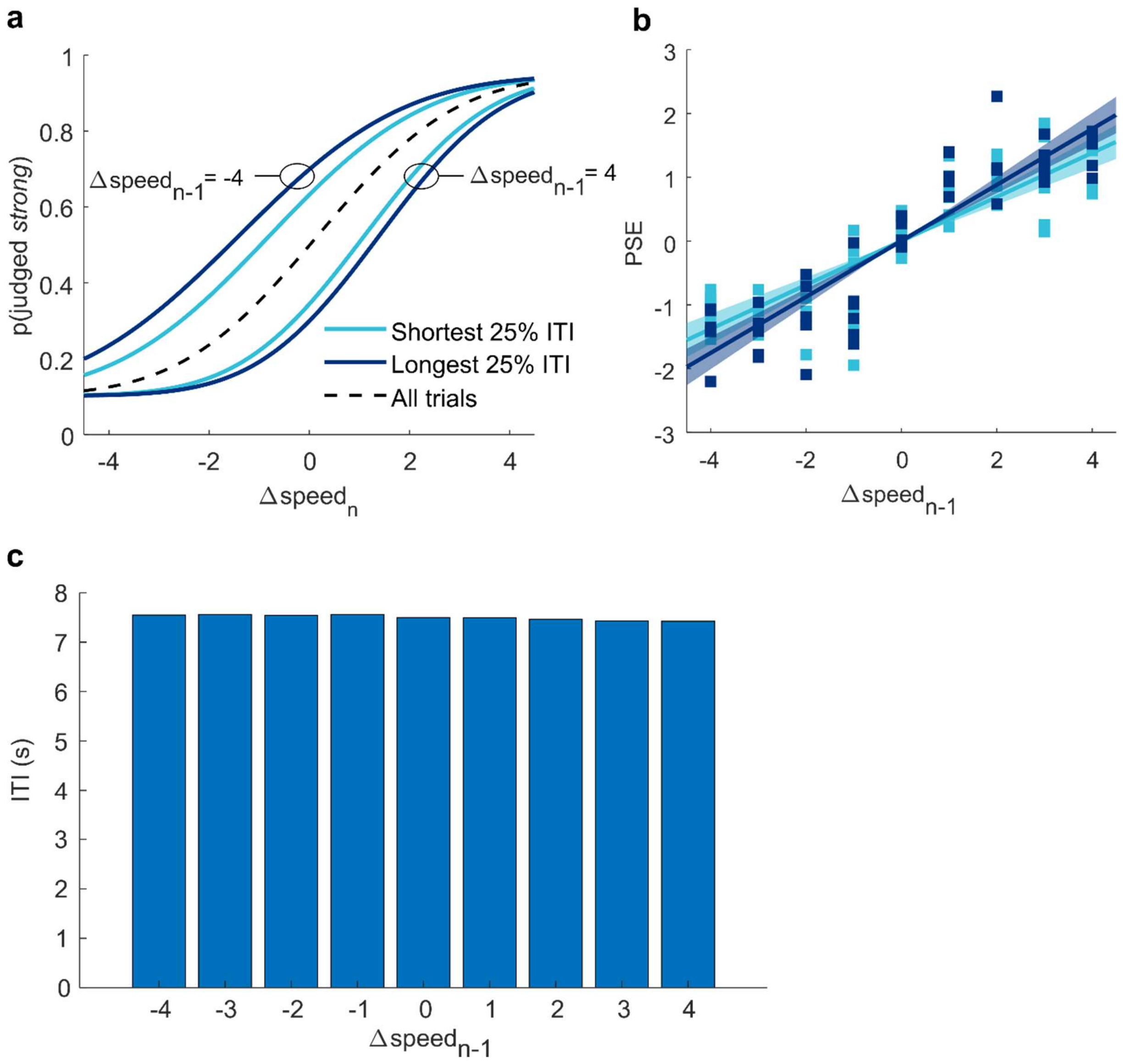

Supplementary Figure S7. Stimulus-dependent bias builds up between trials even when removing incorrect trials and considering only early-session trials. ITI is independent of Δspeed of stimulus *n-1* a. Psychometric curves on trial *n* plotted according Δspeed of stimulus *n-1* and ITI, only using correct choices in trial *n-1* and early session trials (n<150). Dashed line is the average psychometric curve for all trials. **b.** Slope of the regression line fit between bias (PSE) and previous trial Δspeed for all rats, separated for shortest 2 ITI quartiles (light blue) and longest 2 ITI quartiles (dark blue), as in Figure 5c. Shading represents 95% confidence intervals. All trials with incorrect choices in trial *n-1* were removed and only early-session trials were considered (see above). Interaction term between the effects of previous Δspeed and ITI durations on choice *n*, p=0.03 (two-tailed t-test of the regression coefficient). **c.** Median ITI from trial *n-1* to *n*, grouped by Δspeed of stimulus *n-1*. Source data are provided as a Source Data file.

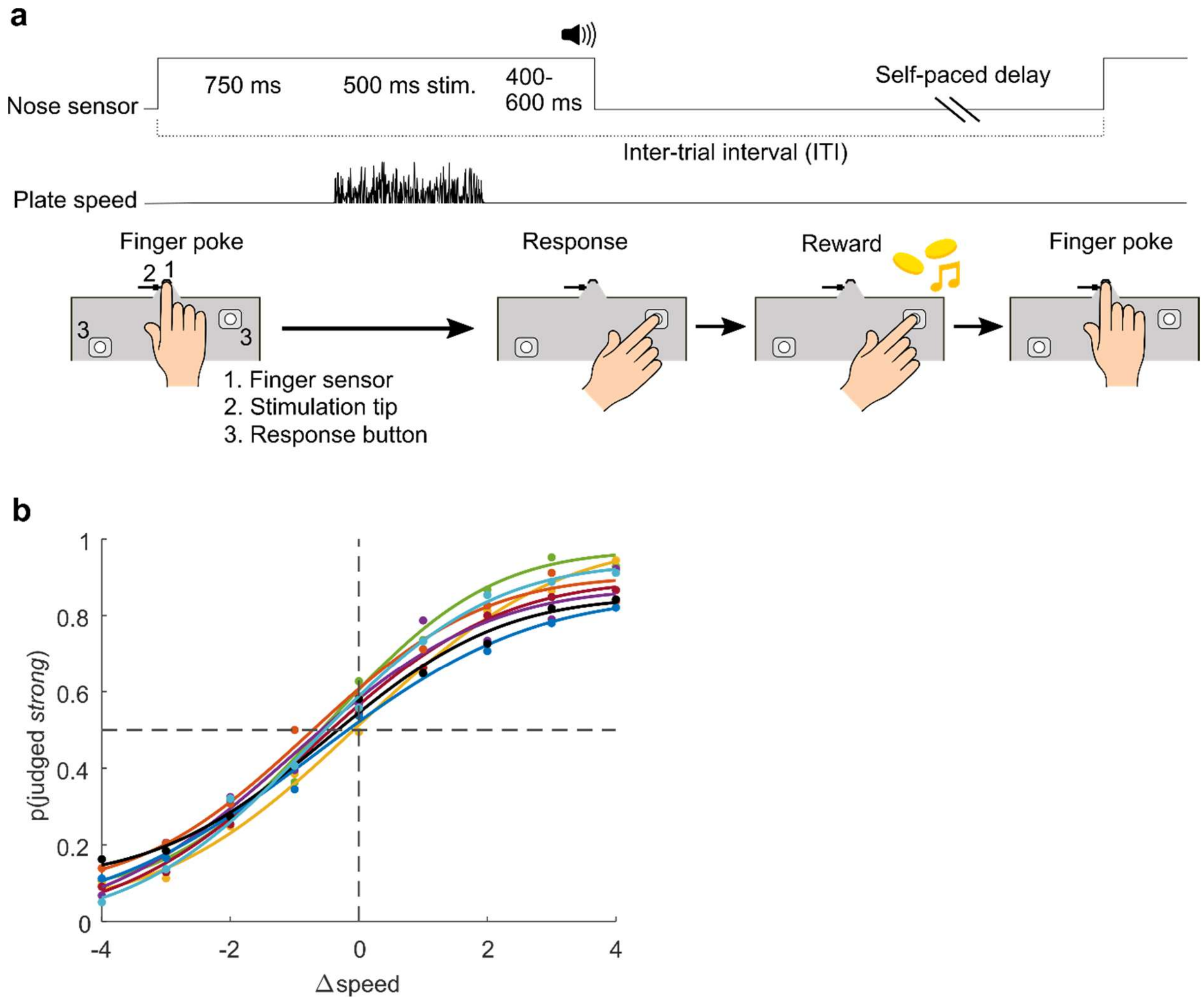

Supplementary Figure S8. Vibrotactile categorization task transferred to human tactile perception. a. Trial configuration. By placing her/his right index finger in the finger poke, the subject triggered a stimulation delivered to the fingertip by means of a vibrating rounded probe. The subject withdrew upon the go cue, and signaled her/his choice side by pressing a button either on the left or the right side. Buttons were asymmetrically placed in order to minimize motor biases (rule was switched between testing sessions). Subjects received feedback (correct/incorrect) on each trial through a computer monitor and headphones. **b.** Psychometric functions fitted to the averaged data of 8 human subjects (approximately 1,500 trials per subject). Source data are provided as a Source Data file.

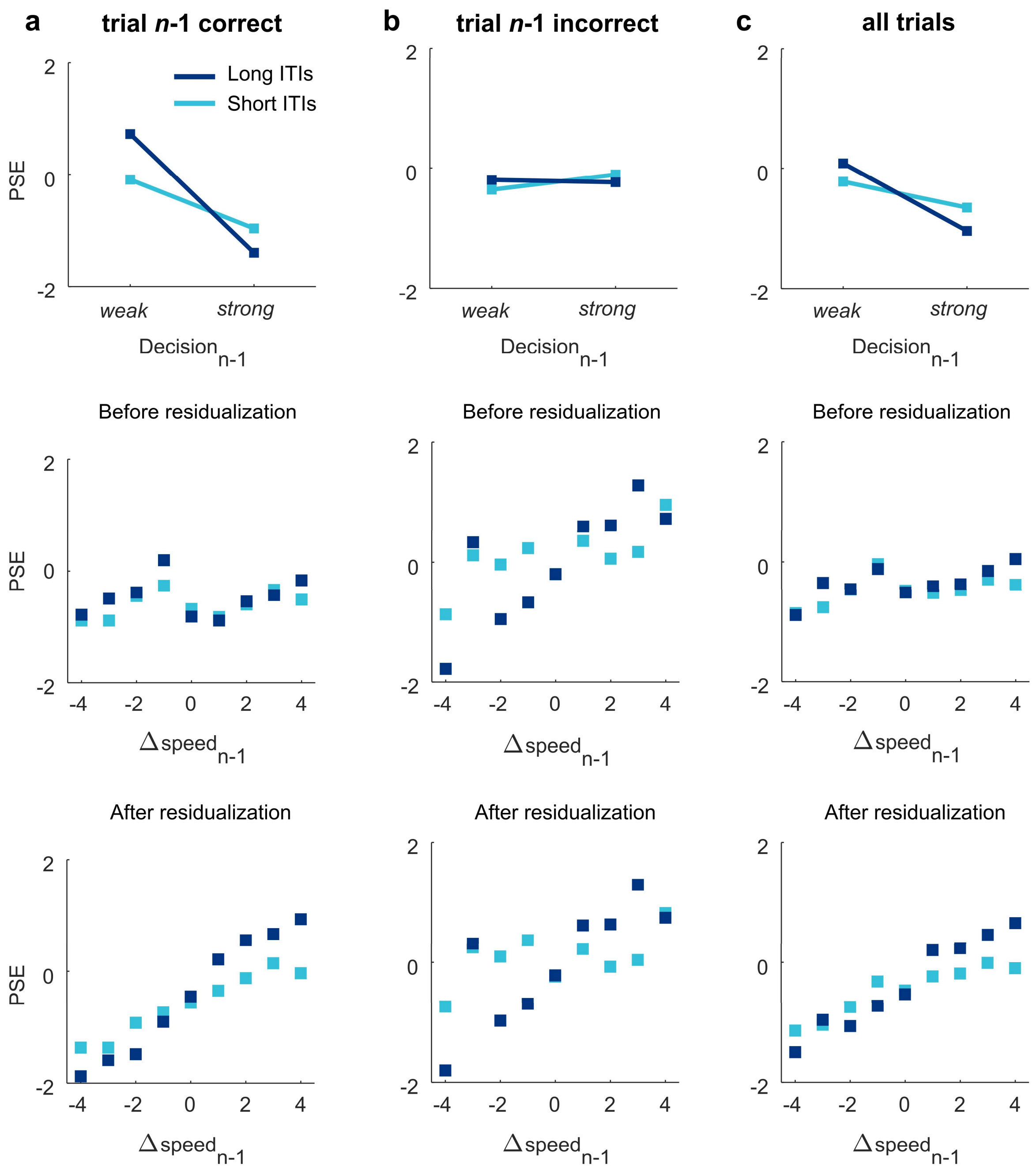

Supplementary Figure S9. Attractive choice bias in humans does not decay over the given ITI durations and does not explain the stimulus history bias after residualization. **a.** Top: Point of subjective equality (PSE) in trial *n* with data grouped by choice in trial *n-1*. Data with *n-1* Δspeed = 0, excluding incorrect trials in trial *n-1*. We found a choice-repetition bias that was slightly larger following long ITIs (dark blue) as compared to short ITIs (light blue). Middle: PSE depending on previous trial Δspeed, following short (light blue) and long (dark

blue) ITIs, excluding incorrect trials in trial *n-1*. Bottom: The choice effects shown in the top panel were subtracted from the PSE values shown in the middle panel. **b.** Top: Point of subjective equality (PSE) in trial *n* with data grouped by choice in trial *n-1*. Data with *n-1* Δspeed = 0, excluding correct trials in trial *n-1*. Choice-repetition bias was absent. Middle: PSE depending on previous trial Δspeed, following short (light blue) and long (dark blue) ITIs, excluding correct trials in trial *n-1*. Bottom: The (minimal) choice effects shown in the top panel were subtracted from the PSE values shown in the middle panel. **c.** Top: Point of subjective equality (PSE) in trial *n* with data grouped by choice in trial *n-1*. Data with *n-1* Δspeed = 0, including both correct and incorrect trials in trial *n-1*. We found a choice-repetition bias that was slightly larger following long ITIs (dark blue) as compared to short ITIs (light blue). Middle: PSE depending on previous trial Δspeed, following short (light blue) and long (dark blue) ITIs, excluding incorrect trials in trial *n-1*. Bottom: The choice effects shown in the top panel were subtracted from the PSE values shown in the middle panel. Source data are provided as a Source Data file.

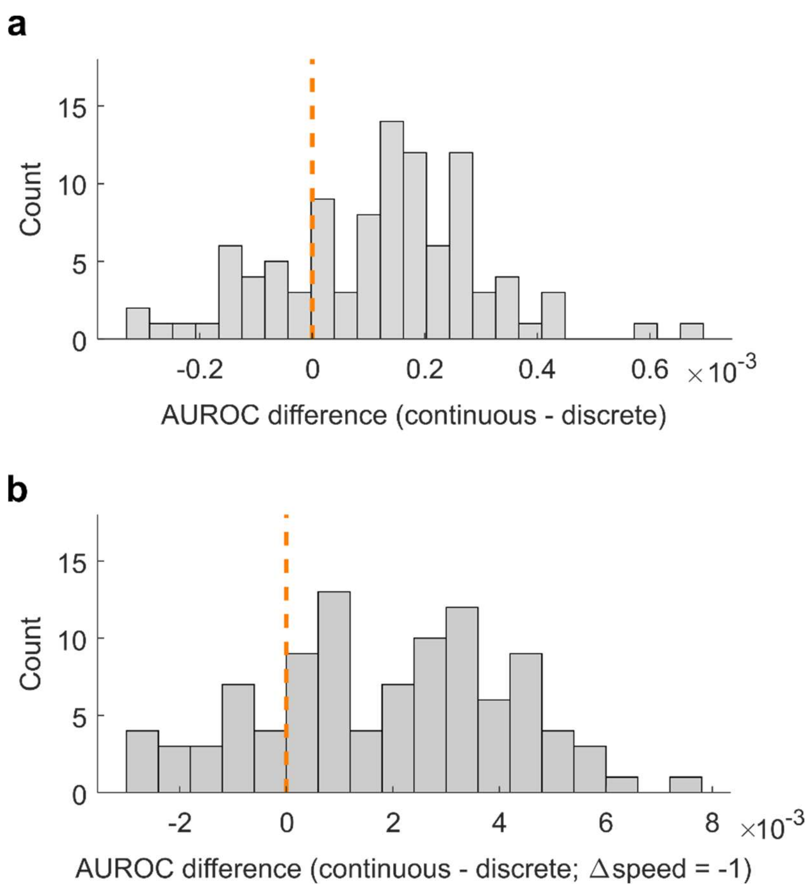

Supplementary Figure S10. Comparative performance of the continuous and discretized history models with Area under the ROC-curve (AUROC). a. Comparison between discrete and continuous model performance considering all trials of the dataset. AUROC was slightly but significantly higher for the continuous model (0.015% greater; p<0.001, two-tailed corrected resampled t-test; Cohen's d = 0.66). **b.** Comparison between discrete and continuous model performance considering exclusively the judgements for Δspeed = -1, for which decisions are most history-driven. This near-center stimulus intensity revealed a larger increase in AUROC (about 0.32%; p<0.001, two-tailed corrected resampled t-test; Cohen's d = 0.8). Brier score was also significantly lower (corrected resampled t-test, p<0.001). Source data are provided as a Source Data file.

	Estimate	SEM	p-value
Intercept	0.0299	0.0093	0.0012
Stimulus_n	0.3273	0.0020	<0.001
Stimulus_n-1	-0.0245	0.0046	<0.001
Reward_n-1	-0.0189	0.0106	0.0754
Stimulus_n-1:Reward_n-1	-0.0461	0.0049	<0.001

Supplementary Table T1. Coefficients of logistic regression analysis including an interaction term between previous stimulus and previous reward. Fitting a GLM with the equation: p(judged *strong*)_n ~ 1 + Stimulus_*n* + Stimulus_*n*-1*Reward_*n*-1, reveals that previous reward interacted weakly with stimulus history. Specifically, the sign of the logistic regression coefficients reflects a decreased influence of the previous stimulus following incorrect trials. P-values are computed from two-tailed t-tests of the regression coefficients. Source data are provided as a Source Data file.

	Estimate	SEM	p-value
Intercept	0.0201	0.0089	0.0239
Stimulus_n	0.3590	0.0039	<0.001
Stimulus_n-1	-0.0713	0.0033	<0.001
Stimulus_n-2	-0.0312	0.0122	0.0108
Stimulus_n-2:ITI_n-1	0.0169	0.0069	0.0136
Stimulus_n-2:ITI_n-2	-0.0185	0.0069	0.0072

Supplementary Table T2. Coefficients of logistic regression analysis with interaction terms between the effect of stimulus *n*-2 and *n*-1 with the adjacent ITIs. GLM with the equation: p(judged strong)_n ~ 1 + Stimulus_n + Stimulus_n-1 + Stimulus_n-2 + Stimulus_n-2:ITI_n-1 + Stimulus_n-2:ITI_n-2. See main text for the explanation of the present analysis. P-values are computed from two-tailed t-tests of the regression coefficients. Source data are provided as a Source Data file.
